# Artificial biofilms establish the role of matrix interactions in staphylococcal biofilm assembly and disassembly

**DOI:** 10.1038/srep13081

**Published:** 2015-08-14

**Authors:** Elizabeth J. Stewart, Mahesh Ganesan, John G. Younger, Michael J. Solomon

**Affiliations:** 1 Department of Chemical Engineering, University of Michigan, 3074 H.H. Dow, 2300 Hayward Street, Ann Arbor, MI 48109; 2 Department of Emergency Medicine, University of Michigan, North Campus Research Complex, 2800 Plymouth Road, Ann Arbor, MI 48109.

## Abstract

We demonstrate that the microstructural and mechanical properties of bacterial biofilms can be created through colloidal self-assembly of cells and polymers, and thereby link the complex material properties of biofilms to well understood colloidal and polymeric behaviors. This finding is applied to soften and disassemble staphylococcal biofilms through pH changes. Bacterial biofilms are viscoelastic, structured communities of cells encapsulated in an extracellular polymeric substance (EPS) comprised of polysaccharides, proteins, and DNA. Although the identity and abundance of EPS macromolecules are known, how these matrix materials interact with themselves and bacterial cells to generate biofilm morphology and mechanics is not understood. Here, we find that the colloidal self-assembly of *Staphylococcus epidermidis* RP62A cells and polysaccharides into viscoelastic biofilms is driven by thermodynamic phase instability of EPS. pH conditions that induce phase instability of chitosan produce artificial *S. epidermidis* biofilms whose mechanics match natural *S. epidermidis* biofilms. Furthermore, pH-induced solubilization of the matrix triggers disassembly in both artificial and natural *S. epidermidis* biofilms. This pH-induced disassembly occurs in biofilms formed by five additional staphylococcal strains, including three clinical isolates. Our findings suggest that colloidal self-assembly of cells and matrix polymers produces biofilm viscoelasticity and that biofilm control strategies can exploit this mechanism.

Bacterial biofilms are cellular communities encapsulated in an extracellular polymeric substance (EPS) of polysaccharides, proteins, and DNA^1^,^2^,^3^. Their viscoelasticity—their tendency to exhibit both a viscous (liquid-like) and an elastic (solid-like) mechanical response—affects fragmentation^4^,^5^ and promotes resilience to shear^5^,^6^,^7^. Biofilms display structural and physicochemical heterogeneity across multiple spatial scales^8^,^9^,^10^,^11^,^12^ and contain pH microenvironments^13^. These heterogeneities are implicated in nutrient and antimicrobial transport within biofilms^2^,^14^, spatial variation in their mechanical properties^15^,^16^, and sociobiology^17^.

In comparison to their planktonic phenotype, the biofilm phenotype of bacteria is structurally and mechanically complex^18^. The cellular densities and concentrations of EPS polymers within biofilms are—in part—regulated by complex pathways of genetic and intercellular signaling^19^,^20^. However, the physical interactions between the cells and the secreted EPS components—interactions that result in a viscoelastic biofilm—are not well understood. There exists an important gap between the understandings of the regulatory, metabolic, and macromolecular synthetic processes that drive biofilm assembly and disassembly and the resultant mechanical characteristics of those biofilms.

Self-assembly—the process by which individual constituents organize into structures as a result of their physical interactions—is a potential contributing factor to biofilm viscoelasticity. In biofilms, there are two self-assembly phenomena that can occur: molecular self-assembly and colloidal self-assembly. Molecular self-assembly describes the associations and structuring among matrix components, while colloidal self-assembly describes the formation of the biofilm itself—the process by which cells combine with the polysaccharide and protein structures of the EPS to produce a viscoelastic material. These colloidal interactions, which arise due to physical forces between suspended particles such as cells^21^ and polymeric structures, can contribute to biofilm morphology and mechanics. For example, it has been suggested that biofilm bacteria can undergo colloidal self-assembly and aggregate due to the depletion interaction of soluble exopolymers^22^,^23^.

In this study, we consider colloidal self-assembly processes in biofilms formed by *Staphylococcus epidermidis*, a representative gram positive bacterium that is one of the most commonly isolated pathogens associated with nosocomial infections^24^. *S. epidermidis* is responsible for the majority of bloodstream infections caused by coagulase-negative staphylococci^25^. Coagulase-negative staphylococci are responsible for 31% of hospital-acquired bloodstream infections in the United States^26^. Biofilm formation is the primary virulence factor for *S. epidermidis*^27^. Thus, identifying the forces that contribute to its microstructure and mechanical properties is important to understanding the disease burden of *S. epidermidis*. Biofilms of this species are viscoelastic, with heterogeneous cellular microstructures ranging from densely packed, disordered bacterial microstructures to low density fractal microstructures of bacterial cells^11^. Its EPS consists predominantly of polysaccharide intercellular adhesin (PIA). PIA contributes to the virulence, resistance, dissemination, and eradication of *S. epidermidis* biofilms^28^.

Here, we study the contribution of physicochemical factors to the elasticity of *S. epidermidis* biofilms by the following: First, through diffusing wave spectroscopy (DWS) measurements of *S. epidermidis* biofilms and their EPS components, we show that the strength (compliance) of the biofilm cannot be explained by the simple summation of the mechanical properties of its individual cellular and polymeric components. Second, we create artificial *S. epidermidis* RP62A biofilms through colloidal self-assembly of planktonic bacteria and abacterial polymeric components, in the absence of any metabolic or regulatory influence. These artificial biofilms match the structure and mechanics of natural *S. epidermidis* RP62A biofilms. The ability to create artificial biofilms that mimic the properties of natural ones shows the role of physical interactions– as opposed to solely genetic processes– in creating biofilm structure and mechanics. Third, we show that the onset of *S. epidermidis* biofilm elasticity coincides with a pH-induced phase instability of the polymeric matrix. This pH-induced phase instability was found to switch the rheological behavior of the artificial biofilm from viscous to viscoelastic, or vice versa. This relationship between EPS phase stability and biofilm mechanical properties was confirmed in natural biofilms of *S. epidermidis* RP62A as well as in additional biofilm-forming staphylococcal strains, including three clinical isolates.

## Results

### Mechanical properties of *Staphylococcus epidermidis* RP62A biofilms and their matrix components

**Figure 1:**
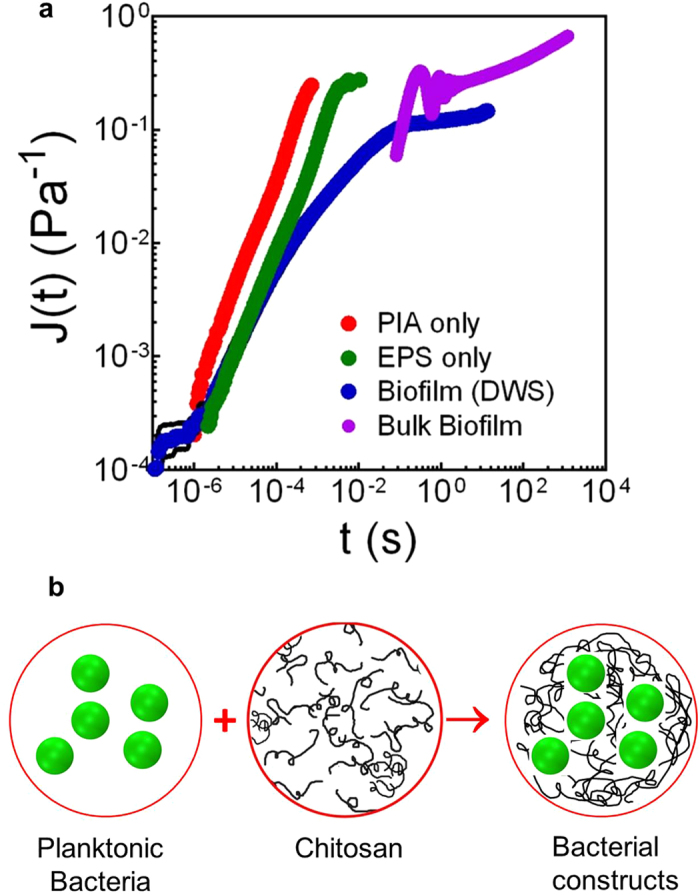
Creep compliances of *S. epidermidis* RP62A biofilm and its polymeric constituents show that cell-polymer interactions strongly contribute to biofilm mechanics. (**a**) Creep compliance, J(t) of PIA(0.016 g/mL), EPS (containing PIA at 0.016 g/mL) and cultured *S. epidermidis* RP62A biofilms. Bulk biofilm data are from^7^. (**b**) Schematic of process to create artificial biofilms from cells and polymers.

Figure 1a compares the linear creep compliance, J(t)—a rheological property indicating viscoelasticity—of a *S. epidermidis* RP62A biofilm with that of its PIA (the primary extracellular polysaccharide), and its EPS (all the extracellular matrix materials). J(t), the creep compliance, is the amount of strain per unit stress that a substance deforms when a constant shear stress is applied. Measurements were made using DWS (c.f. Methods) and are plotted for the case of PIA, EPS, and cultured biofilm. Mechanical rheometry data for cultured *S. epidermidis* RP62A biofilms^7^ is also plotted. The biofilm mechanical rheometry results are congruent with the DWS measurements, which are reported for the particular case of colloidal probes with diameter comparable to that of the *S. epidermidis* cells (c.f. Methods for additional discussion).

The creep compliance of *S. epidermidis* biofilms is characteristic of a viscoelastic material, because the response is comprised of an elastic (solid-like) contribution, as seen by a rapid increase to near constant value, followed by a viscous (liquid-like) contribution, identified by the steady increase in compliance at longer times (Fig. 1a). However, the extracellular polymers synthesized by *S. epidermidis*—either PIA or the entire acellular EPS^28^—show only a viscous response, as seen by their linear increase in J(t). The qualitative difference between the creep compliance of the biofilm and of the PIA and EPS is contrary to the idea that exopolymers are the prime determinant of biofilm viscoelasticity^18^,^29^. Hence, comparison of the mechanics of different components of the biofilm indicates neither PIA nor EPS alone have the mechanical behavior of a natural *S. epidermidis* biofilm.

Thus, Fig. 1a shows the significant difference between the mechanics of a natural *S. epidermidis* biofilm and its individual matrix components (PIA and EPS). Specifically, the *S. epidermidis* RP62A EPS polymers alone are insufficient to generate the mechanics of a mature *S. epidermidis* RP62A biofilm. However, staphylococcal PIA is known to exhibit self-associations as a function of pH^30^; these self-associations can create colloidal, phase-separated structures that interact with nearby cells. Therefore, we seek to understand if such pH-mediated association of biofilm exopolymers could immobilize bacterial cells in the EPS and thereby generate biofilm viscoelasticity.

### Self-assembly of biofilm-like constructs

We hypothesize that biofilm viscoelasticity emerges from the colloidal self-assembly of its constituent cellular and polymeric components—cells, proteins, and polysaccharides. This self-assembly induces self-organization of volumes containing thousands of cells into viscoelastic soft matter. Biofilm morphology and viscoelasticity are then the consequence of processes akin to those of attractive colloids, whose self-organization yields heterogeneous, viscoelastic structures^18^,^31^.

To test this hypothesis, planktonic *S. epidermidis* bacterial cells and abacterial proxies for the extracellular polysaccharides, proteins, and DNA were combined to create artificial biofilms (Fig. 1b). The abacterial proxies are not synthesized by *S. epidermidis.* Because the pH of *S. epidermidis* biofilms is reported to vary from 4.5–7.5^32^,^33^,^34^, and because the associative interactions in staphylococcal polysaccharides depend on pH^30^, the pH of the constructs was carefully controlled. The use of abacterial components eliminates bacterial metabolic or synthetic activity as an explanation of the artificial biofilm morphology and mechanics that we report. Creating artificial biofilms furthermore allows for independent control of the concentration of cellular and matrix components as well as the properties of the solvent environment, such as pH. These artificial biofilms offer a new way to identify the role of colloidal interactions in mediating biofilm mechanical properties.

**Figure 2:**
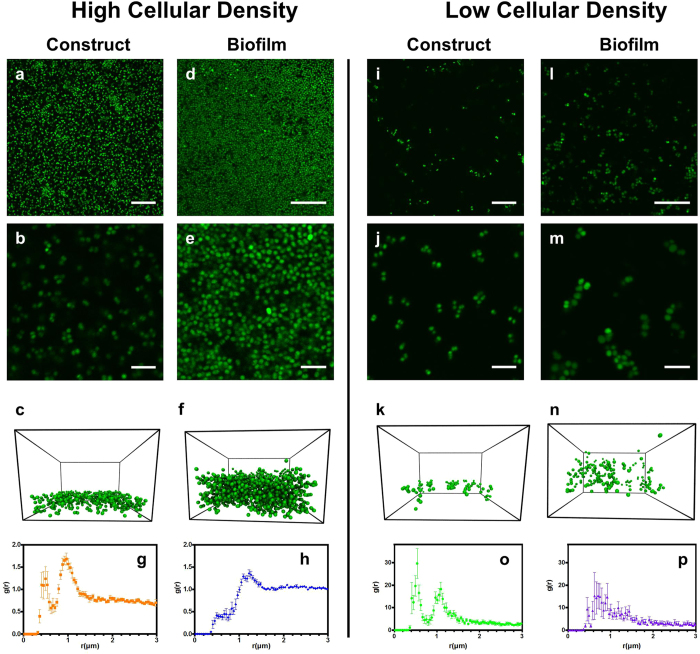
Microstructure of high and low cellular density *S. epidermidis* RP62A-chitosan constructs and biofilms shows that the colloidal self-assembly of cells and polymeric constituents leads to biofilm-like cellular organization. Left half compares high cellular density constructs (pH = 4.3) and high-density biofilms; right half compares low cellular density constructs (pH = 5.3) and low-density biofilms. First and second rows: CLSM images of (**a**,**b**) high cellular density bacteria-chitosan constructs; (**d**,**e**) high-density biofilms, (**i**,**j**) low cellular density constructs, and (**l**,**m**) low-density biofilms. Third row (**c**,**f**,**k**,**n**): volume renderings of bacterial positions. Fourth row: Radial distribution function, g(r), of (**g**) high cellular density constructs, (**h**) high-density *S. epidermidis* RP62A biofilms, (**o**) low cellular density constructs, and (**p**) low-density *S. epidermidis* RP62A biofilms. Scale bars, 20 μm (**a,d,i,l**) and 5 μm (**b,e,j,m**).

**Figure 3:**
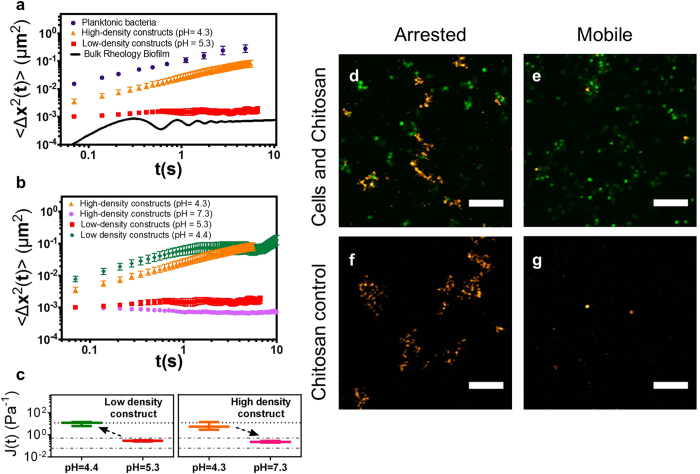
pH-effects on chitosan stability control whether cellular dynamics are characteristic of planktonic or biofilm phenotypes in artificial *S. epidermidis* RP62A biofilms. (**a**) Mean squared displacement, 〈Δx^2^(t)〉, of planktonic *S. epidermidis* RP62A bacteria and bacteria in high (pH = 4.3) and low (pH = 5.3) cellular density *S. epidermidis* RP62A constructs compared with biofilm 〈Δx^2^(t)〉 inferred from ref. 7. (**b**) 〈Δx^2^(t)〉 of high cellular density constructs before (pH = 4.3) and after (pH = 7.3) pH is increased, and low cellular density constructs before (pH = 5.3) and after (pH = 4.4) pH is decreased. (**c**) J(t), at t = 1 s, of high and low-density constructs before and after pH changes; arrows indicate the direction of pH change. The upper dotted line is the creep compliance, J(t), of planktonic cells. The dotted dashed lines bound the J(t) observed for *S. epidermidis* RP62A biofilms^7^. (**d**) CLSM image of cells and 0.05 wt. % chitosan at pH = 5.3. (**e**) CLSM image of cells and 0.3 wt. % chitosan at pH = 4.3. (**f**) 0.05 wt. % chitosan in tryptic soy broth with 1 wt. % added glucose (TSB_G_). (**g**) 0.3 wt. % chitosan in TSB_G_. Scale bars, 10 μm (**d**–**g**).

We examined 18 different construct assembly conditions, in which cellular concentrations, matrix material concentrations and pH were varied (c.f. Methods). Figures 2, 3 report the findings for the cases in which: i) the EPS of natural *S. epidermidis* has been replaced with chitosan (a common polysaccharide that differs from PIA only in its glycosidic linkages and its degree of acetylation) and ii) the construct microstructures match those of natural *S. epidermidis* biofilms. When constructs were made using an EPS of chitosan, proteins, and DNA, the constructs were found to be mechanically similar to those made from a matrix of chitosan alone (Supplementary Fig. S1). Thus, for simplicity, results for constructs produced with chitosan alone are presented.

### Biofilm-like microstructure of bacterial-chitosan constructs

*S. epidermidis* biofilms are comprised of both high and low cellular density phenotypes^11^. Artificial *S. epidermidis* RP62A biofilms equivalent to the high cellular density phenotype were self-assembled using an initially dilute suspension of cells and chitosan at 0.3 wt. % and pH of 4.3 (Fig. 2a,b). The number density of cells was 0.162 ± 0.001 cells/μm^3^, which was similar to the high-density phenotype of natural biofilms of this species (0.2 cells/μm^3^ or greater)^11^. A 3D confocal laser scanning microscopy (CLSM) rendering of bacterial centroids within the artificial biofilm shows that the cells span the volume heterogeneously, just as in natural biofilms^8^,^9^,^10^ (compare Fig. 2a–f).

The cellular radial distribution function, g(r)—the probability of finding a cell at a distance r from a reference bacterium—quantifies the local cellular organization of the biofilm^11^. The high cellular density artificial biofilm g(r) is characteristic of a densely packed, disordered microstructure (Fig. 2g). The peak at r = 0.5 μm is due to cell division. The primary peak at r = 1.0 μm matches the primary g(r) peak of natural *S. epidermidis* RP62A biofilms of the high-density phenotype (Fig. 2h).

Artificial biofilms equivalent to the low-density phenotype of natural *S. epidermidis* RP62A biofilms (local number density = 0.017 ± 0.002 cells/μm^3^) were created from initially dilute planktonic cells and chitosan at 0.05 wt.% and pH of 5.3. These assemblies were qualitatively more clustered than the high-density biofilms, and had cellular densities that matched low-density phenotype natural *S. epidermidis* biofilms (0.06 cells/μm^3^ or less). They displayed open structures (Fig. 2i,j) and were spatially heterogeneous (Fig. 2k) like their natural equivalents (Fig. 2l,m,n). The g(r) peak values of the artificial and natural biofilms differ only by 22% (Fig. 2o,p). Thus, the complete range of cellular microstructures present in the biofilm phenotype of *S. epidermidis* can be artificially created, in the same growth environment, by mixing planktonic staphylococci and chitosan at particular concentrations and pH.

### Cellular mobility and viscoelasticity of bacterial-chitosan artificial biofilms

Figure 3a reports the mean-squared displacement 〈Δx^2^(t)〉 (MSD), of cells within the different bacterial-chitosan constructs studied. The MSD of individual bacteria within natural or artificial biofilms quantifies their mobility and reveals viscoelastic features of the environment surrounding the cells. When in a purely viscous medium, the MSD increases linearly with time over the course of extended observation, i.e., cells in a viscous medium will perform Brownian walks over time. In contrast, for an elastic medium, the MSD remains fixed, i.e., cells will tend to remain on average in the same position. Thus, the MSD characterizes biofilm rheology. Specifically, the mean-squared displacement is proportional to the creep compliance^35^:


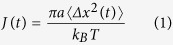


Where, *J(t)* is the creep compliance, *a* is the radius of the cell, and k_B_ and T are the Boltzmann constant and temperature, respectively. We compare the creep compliance of the constructs, as computed from the MSD and eqn. (1), with that of *S. epidermidis* biofilms reported by Pavlovsky *et al.*^7^.

Cells within high-density artificial biofilms, are less mobile than planktonic bacteria, but more mobile than cells within natural *S. epidermidis* biofilms (Fig. 3a). The near-linear increase in their MSD is characteristic of a viscous suspending medium (Fig. 3a, Supplementary Videos 1, 2), and differs from the MSD of natural *S. epidermidis* biofilms which exhibit a plateau (Fig. 3a) that is characteristic of elastic behavior. (The MSD of natural biofilms was obtained from their creep compliance following equation (1) and the mechanical rheometry data of Pavlovsky *et al.* in Fig. 1a.) Although the microstructure of this artificial biofilm matches the high-density biofilm phenotype, the cellular mobility and construct mechanics are significantly different from natural *S. epidermidis* RP62A biofilms.

Cells in the low density construct on the other hand are nearly arrested, as exhibited by their nearly time independent MSD. This time-independent mobility implies that the low-density artificial biofilms are elastic. The cellular localization is consistent with an elastic modulus ∼2.6 Pa (Fig. 3a, Supplementary Video 3). Natural *S. epidermidis* biofilms produced under similar conditions have an elastic modulus of ~3.7 Pa^7^. The low-density construct and the natural biofilm moduli are nearly equivalent, especially when considered relative to the ~10^4^ variability reported for natural *S. epidermidis* biofilms^5^.

Cells in the low-density artificial biofilm are therefore localized, consistent with a viscoelastic biofilm, while cells in the high-density artificial biofilm are not localized. This change in cellular mobility could be a consequence of their different cellular concentrations, the different chitosan concentrations, or the different pH values. Of these variables, pH was found to control the mechanics of the artificial *S. epidermidis* biofilms. Specifically, when the pH of the high-density artificial biofilms was increased from 4.3 to 7.3, the cellular MSD changed from viscous diffusion to elastic localization (Fig. 3b, Supplementary Video 4). Analogously, when pH of the low-density artificial biofilms was decreased from 5.3 to 4.4, the mobility of the arrested cells increased and approached diffusive behavior (Fig. 3b, Supplementary Video 5). Thus, as pH is increased, the J(t) of low- and high-density artificial biofilms approximate that of natural *S. epidermidis* biofilms (Fig. 3c). As pH is decreased, J(t) of both high- and low-density artificial biofilms approximate that of planktonic bacteria (Fig. 3c). Thus, increasing or decreasing the pH affects whether or not cells are mobile or immobile in the artificial biofilms.

Neither chitosan concentration (Fig. 3c) nor cellular concentration (Supplemental Figure S2) is critical to the mechanical behavior observed. For example, as shown in Fig. 3c, at a pH of 4.4 constructs formed with both 0.3 wt. % chitosan and 0.05 wt. % chitosan contain cells that diffuse with similar mobility. Thus, the pH environment of the constructs is responsible for changing the mechanical behavior of artificial *S. epidermidis* RP62A biofilms.

### Mechanism of the pH effect on bacterial mobility in artificial biofilms

Visualization of chitosan within the arrested and mobile constructs reveals the effect of pH on chitosan structure within the artificial biofilms (Fig. 3d,e). In the artificial biofilms formed in TSB_G_ at pH = 5.3, chitosan formed a stringy network between the bacterial cells that visibly spanned the image volume (Fig. 3d). However, in constructs formed at pH = 4.3 in TSB_G_, the stringy chitosan network was absent indicating uniform dispersal of the polymer (Fig. 3e). The formation of a stringy network is representative of an unstable phase, where the polymer visibly comes out of solution; while, the absence of such a network indicates a stable phase where the polymer is homogeneously dispersed in the solution. Control experiments of chitosan solution in TSB_G_ without cells (Fig. 3f,g) further confirm the pH effect.

Figures 2, 3 support the central role of self-assembly in producing biofilm structure and dynamics because an artificial biofilm can be created with microstructure and mechanics that match those of natural *S. epidermidis* biofilms. Figure 3d–g suggests that these structures and mechanics are a consequence of the colloidal self-assembly of the cellular and polymeric components of the biofilm. Specifically, chitosan phase instability (Fig. 3d) is coincident with dynamical arrest of the *S. epidermidis* cells (Fig. 3b). The pH-induced phase instability of chitosan drives the generation of viscoelasticity in the artificial biofilms produced from planktonic cells and chitosan.

### pH-induced phase instability of chitosan and *S. epidermidis* biofilm EPS

**Figure 4:**
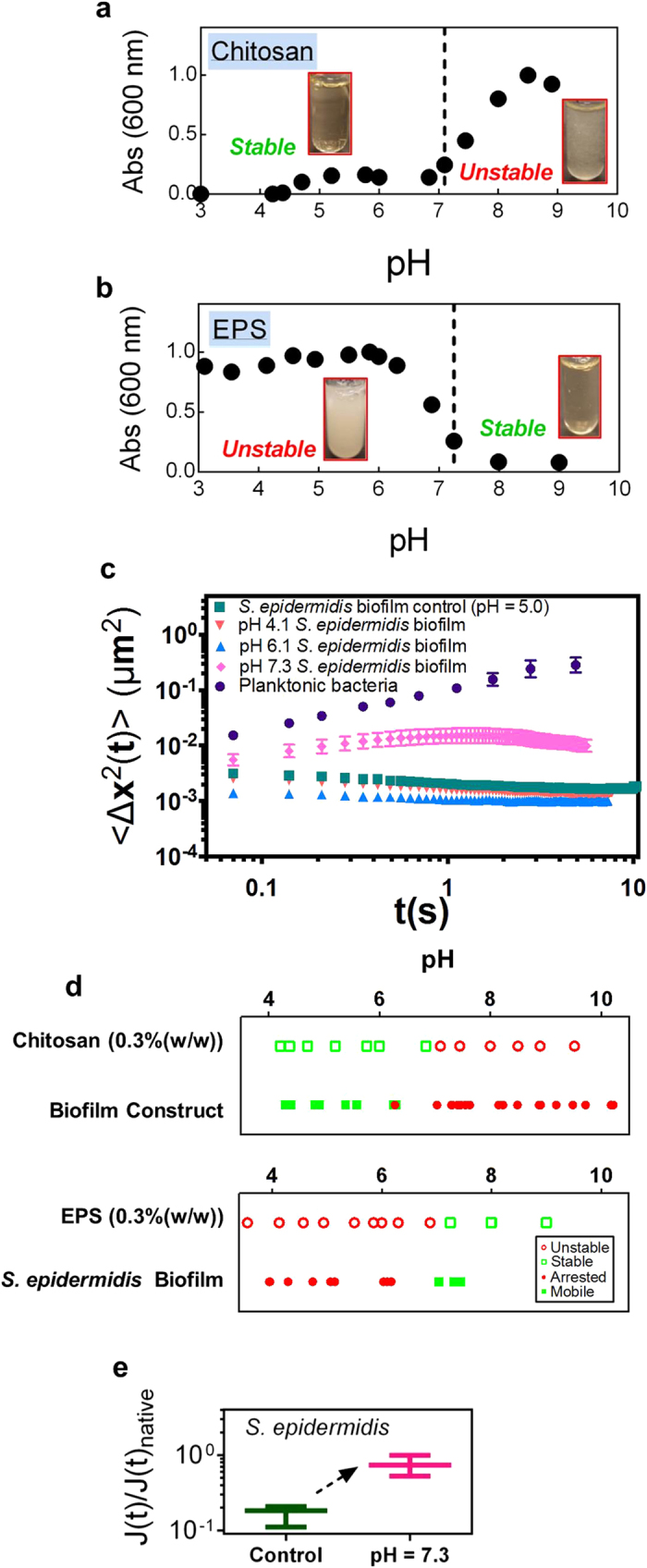
The stability of EPS, as mediated by pH, controls the microrheology of natural *S. epidermidis* RP62A biofilms, consistent with the behavior of chitosan in artificial biofilms. Absorbance versus pH of 0.3 wt. % chitosan (**a**) and 0.3 wt. % *S. epidermidis* RP62A biofilm EPS (**b**) in TSB_G_. (**c**) Mean squared displacement, 〈Δx^2^(t)〉 of *S. epidermidis* RP62A planktonic bacteria, 18-hour biofilm, and 18-hour biofilms with pH adjusted to 4.1, 6.1, and 7.3. (**d**) Comparison of 0.3 wt. % chitosan stability and the mobility of 0.3 wt. % bacterial-chitosan constructs, as well as the stability of 0.3 wt. % EPS and the mobility of 18-hour *S. epidermidis* RP62A biofilms at pH 4–10. (**e**) Normalized creep compliance, J(t), at t = 1 s, of *S. epidermidis* RP62A biofilms after increasing the pH to 7.3.

From the findings for artificial biofilms (Figs 2, 3), we infer that association and phase instability of the biofilm’s EPS, as potentially induced by pH change, could be correlated with the assembly and disassembly of natural *S. epidermidis* biofilms. To test this proposition, we check if phase instability of natural *S. epidermidis* biofilm EPS occurs as for chitosan in the artificial biofilms. Here phase instability is evaluated with an absorbance measurement because phase instability generates turbidity. Figure 4a,b compare the pH dependent absorbance of chitosan and *S. epidermidis* EPS. Consistent with the results of Fig. 3d–g, chitosan showed a significant increase in turbidity at pH ~ 7. This pH marks a transition from a stable, low absorbance phase at low pH to an unstable, high absorbance phase at high pH. The absorbance measurements for the *S. epidermidis* EPS also show that its phase stability is controlled by pH. In this case, however, the effect of pH on *S. epidermidis* EPS stability is reversed relative to chitosan; the EPS is stable at high pH and unstable at low pH, with a transition at pH ~ 7. This reversal suggests that a change in natural *S. epidermidis* biofilm pH from low to high will result in loss of biofilm viscoelasticity.

### Application of findings to soften *S. epidermidis* biofilms

Figure 4c tests whether an increase in the pH of the *S. epidermidis* RP62A biofilm will increase the mobility of the cells within the biofilm. This prediction is consistent with: (i) the fact that chitosan phase stability controls cellular mobility in the chitosan constructs; and (ii) that EPS phase stability increases at pH > 7 as shown in Fig. 4b. When the pH of a natural *S. epidermidis* RP62A biofilm was increased from 5.0 to 6.1, the biofilm bacteria remained arrested (Fig. 4c); however, when the pH was increased further to 7.3, beyond the Fig. 4b inflection in EPS phase stability, cellular mobility increased and approached that of planktonic bacteria (Fig. 4c, Supplementary Videos 6, 7). This change in biofilm mechanics occurs at a pH at which the EPS absorbance is low, consistent with thermodynamic stability of the EPS. Correspondingly, when the pH of the natural biofilm was decreased from 5.0 to 4.1, the bacterial cells remained arrested, consistent with the observation that the EPS was unstable at those pH values.

Figure 4d compares the pH-mediated behavior of chitosan with that of the artificial biofilms and the pH behavior of the EPS with that of the natural *S. epidermidis* RP62A biofilms. Chitosan becomes unstable when pH is 7.1 or higher, consistent with the transition from a mobile to an arrested artificial biofilm when its pH is increased to 5.6–7.3 (Fig. 4d). When EPS is the matrix, it transitions between stable and unstable phases at pH 7. High-density *S. epidermidis* RP62A biofilms transition from an arrested state to a mobile state when pH is increased to 6.2–7.0. The direction of the change in matrix phase stability (i.e. absorbance) and viscoelasticity (i.e. cellular mobility) with pH is consistent in every case. Interestingly, the increase in cellular mobility within the natural *S. epidermidis* biofilm at pH 7.3 (Fig. 4c) represents an instance of biofilm softening (Fig. 4e).

**Figure 5:**
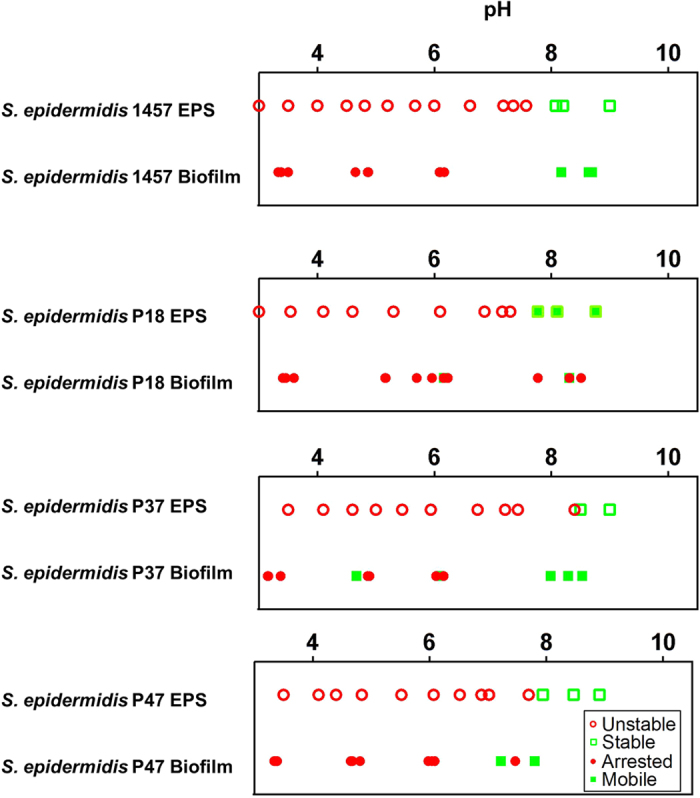
pH controls EPS stability and biofilm mobility in *S. epidermidis* 1457 and *S. epidermidis* clinical isolates P18, P37, and P47. Comparison of the pH mediated phase stability and cellular mobility of the EPS and the respective 18-hour biofilm of the different *S. epidermidis* strains studied; the pH ranges from 4–10.

Furthermore, similar pH changes induce softening of biofilms produced by other *S. epidermidis* strains. Biofilms formed by *S. epidermidis* 1457 and three *S. epidermidis* clinical isolates—*S. epidermidis* P18, P37, and P47 from Sharma *et al.*^36^—were subjected to pH change. The stability of their EPS matrix was characterized by an absorbance measurement. In *S. epidermidis* 1457, a common laboratory strain^37^, the transition from an unstable to a stable extracellular matrix occurred between pH 7.6–8.1. The pH-induced change in mobility of the *S. epidermidis* 1457 biofilm corresponded with this transition (Fig. 5). In the three clinical isolates, we observed increased phase stability of the matrix materials at high pH. Additionally, increased cellular mobility—characteristic of biofilm softening—occurred in all three clinical isolates at high pH (Fig. 5). The exact pH of the matrix stability transition does not align as closely with the mobility transition for the three clinical isolates as it does for the two common laboratory strains (*S. epidermidis* RP62A and 1457). Nevertheless, the correlation of the phase instability and mobility of the clinical isolates is consistent with the results for the two laboratory strains.

### Application of findings to soften *S. aureus* biofilms

**Figure 6:**
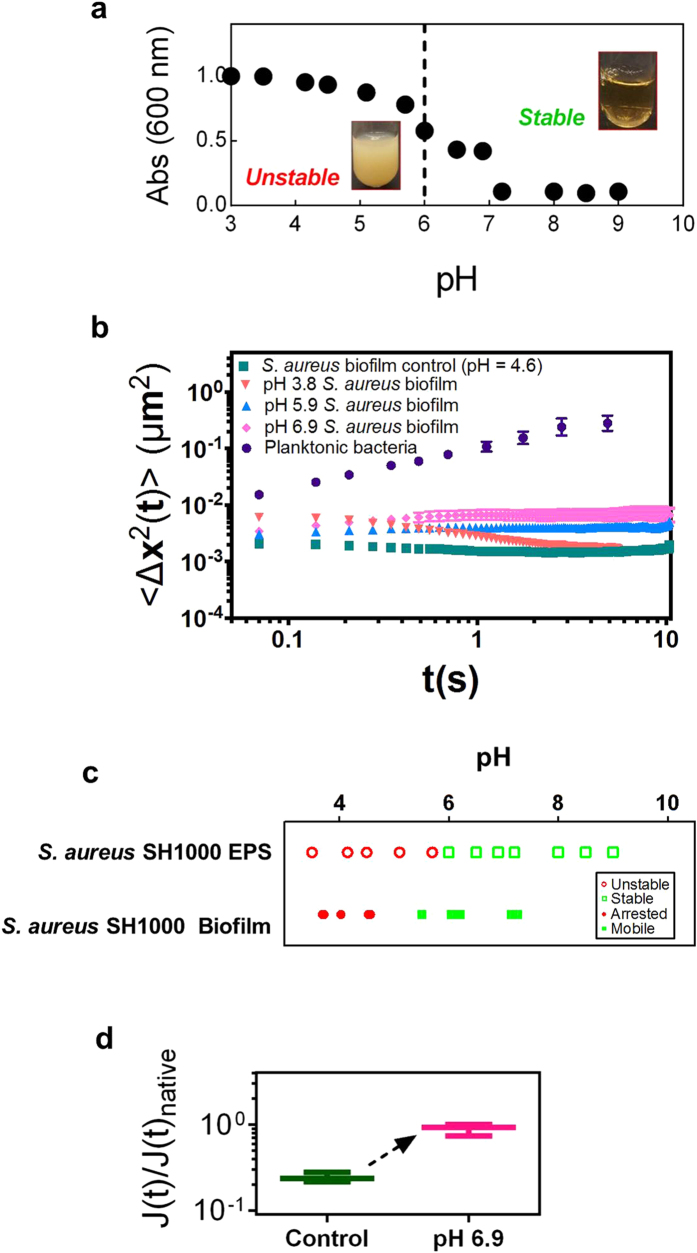
pH controls EPS stability and biofilm mobility in *S. aureus* SH1000 (**a**) Absorbance versus pH of 0.3 wt. % *S. aureus* SH1000 biofilm EPS in TSB_G_. (**b**) Mean squared displacement, 〈Δx^2^(t)〉, of planktonic cells, *S. aureus* 18-hour biofilm, and 18-hour biofilms with pH adjusted to 3.8, 5.8, and 6.9. (**c**) Comparison of the stability of 0.3 wt. % *S. aureus* SH1000 EPS and the mobility of 18-hour *S. aureus* SH1000 biofilms at pH 4–10. (**d**) Normalized creep compliance, J(t), at t = 1 s, of *S. aureus* SH1000 biofilms after increasing the pH to 6.9.

*Staphylococcus aureus* is a biofilm forming pathogenic species associated with blood stream infections^38^. *S. aureus* secretes a N-acetylglucosaminoglycan that closely resembles the PIA of *S. epidermidis* biofilms^39^. If the phase stability of the PIA-rich EPS controls cellular mobility in natural *S. epidermidis* biofilms, then a similar response should be observed in *S. aureus*. To test this, we probed the pH dependent absorbance of *S. aureus* EPS in TSB_G_. Figure 6a shows that at pH ~ 6, *S. aureus* EPS transitioned from an unstable to a stable phase. Correspondingly, when *S. aureus* biofilm pH was increased from 4.6 to 6.9, the MSD of the cells within the biofilm increased by up to a factor of four (Fig. 6b, Supplementary Videos 8, 9). Thus, thermodynamically stable, non-turbid EPS and softened *S. aureus* SH1000 biofilms (Fig. 6c) occur at similar pH. The correlation of EPS phase stability and biofilm viscoelasticity thus holds for two staphylococcal species.

## Discussion

This study has found that artificial staphylococcal biofilms of bacteria and chitosan match the structure (Fig. 2) and microrheology (Fig. 3) of natural biofilms when the pH of the artificial biofilm is such that the matrix is an unstable phase (Figs 3d). The matrix phase instability is a consequence of associations driven by molecular self-assembly of the polymers. This matrix phase instability triggers the colloidal self-assembly of the cells and polymers into viscoelastic biofilms. This finding was applied to soften natural biofilms by exploiting the pH dependent phase stability of the biofilm’s EPS polymers (Figs 4, 5, 6). Thus, these findings establish colloidal self-assembly as a factor in biofilm formation and dispersal. The correlation between the EPS phase stability and the mechanical properties of the *S. epidermidis* biofilms indicates that the viscoelasticity of these biofilms is not just an additive effect of the individual mechanics of polymers and cells, but instead includes cross contributions generated by associations among EPS polymers, cells, and the solvent environment.

The connection between pH and *S. epidermidis* EPS phase stability could facilitate microbial survival. That is, biofilm spatial and temporal pH gradients may be involved in the formation or breakdown of biofilm elasticity through changes in the EPS stability. pH variation within biofilms can be a consequence of bacterial metabolism^40^, while the composition of the EPS is governed by genetic regulation. Thus, the role of pH in mediating biofilm elasticity presents an interesting potential coupling between metabolism (that changes the pH microenvironment within biofilms), genetic regulation (that mediates EPS composition) and biofilm mechanical properties. Additionally, these couplings could extend to biofilm disassembly and dispersion.

The sensitivity of biofilm elasticity to pH suggests strategies to control and eradicate biofilms through pH-induced softening. For *S. epidermidis,* we have shown that disrupting the physicochemical factors that generate phase instability of the EPS is a potential strategy for biofilm remediation. Specifically, staphylococcal biofilm control strategies could exploit the increase in cellular mobility that occurs between pH 5–8. Current biofilm removal strategies include the use of matrix degrading enzymes such as proteases, DNAse, Dispersin B and disruptive agents such as chelators^41^. Digestive enzymes are known to target specific components of the EPS, while the method presented here targets the collective biofilm EPS. Thus, the method presented here yields a similar effect as the digestive enzymes and introduces a new strategy for developing biofilm control techniques.

Mechanistic understanding of why pH affects *S. epidermidis* matrix phase stability is a direction for future work. The mechanism of molecular self-assembly leading to the matrix phase transition needs to reconcile two observations. First, the pH dependence of the chitosan phase instability reported in Fig. 4a is specific to the growth media—TSB with 1% added glucose—in which the bacterial-constructs were produced. Second, the pH dependence of the phase behavior of the chitosan and *S. epidermidis* RP62A EPS in this media is antipodal; chitosan is unstable at pH > 7; *S. epidermidis* RP62A EPS is unstable at pH < 7. The sensitivity of the phase instability to media is expected because its constituents—glucose, salt (sodium chloride, potassium diphosphate), and peptides (tryptone and soytone)—can affect association and stability of the polysaccharide by interacting with hydrophobic and charged regions of this macromolecule. Furthermore, the chitosan used to produce the artificial biofilms is highly deacetylated (DA ~ 75%). The resulting amino groups (pKa ~ 6.5) confer stability at low pH. The staphylococcal polysaccharide, PIA, on the other hand, is much less charged (DA ~ 20%); its instability at low pH (c.f. Fig. 4b) points to complexation of PIA with negatively charged species of the EPS—proteins and extracellular DNA.

The present investigation of biofilm softening was observed for staphylococcal strains grown in 8-well culture chambers. Further investigation to identify if the pH induced softening extends to other microbial species and to other culture conditions is needed to assess the breadth of the findings. Extension of the work to gram-negative bacteria (e.g. *Pseudomonas aeruginosa* or *Klebsiella pneumoniae*^42^) would be of particular interest. Furthermore, investigation of biofilm softening in other growth environments, such as flow-cells, flasks, or culture chambers, is also warranted.

This study suggests a two-step procedure to design techniques for biofilm softening. First, identify factors that change the phase stability of the biofilm’s extracellular matrix. Second, change the biofilm environment to a condition at which the matrix forms a stable, non-turbid phase. Such a condition should result in biofilm softening due to matrix disassembly. This prediction follows from the finding that biofilm viscoelasticity can be artificially created through controlling the interactions that generate colloidal self-assembly of cells and matrix polymers.

## Materials and Methods

### Bacterial strains and growth media

*S. epidermidis* RP62A(ATCC 35984) was used for studies of biofilm and matrix mechanics as well as the creation of the artificial biofilms. Additional strains of biofilm forming *S. epidermidis* were used: *S. epidermidis* 1457—a common laboratory used biofilm former^37^—and three biofilm forming clinical isolates of *S. epidermidis:* P18, P37, and P47 from Sharma *et al.*^36^. The three clinical isolates were chosen from a library of 54 patient samples reported in Sharma *et al.*^36^ based on their biofilm forming capability. Methods to identify biofilm forming clinical isolates from Sharma *et al.*^36^ are presented in Supplementary Information. *S. aureus* SH1000 (provided by B.R. Boles) was used as a representative strain of *S. aureus.* All strains were streaked on tryptic soy agar plates. Colonies were cultured using tryptic soy broth with 1 wt. % glucose (TSB_G_) as the growth media.

### Biofilm growth methods and bacterial culture conditions (in order of appearance in results)

#### Extraction and quantification of biofilm polymers

*S. epidermidis* biofilms were cultured and the extracellular polymeric substances (EPS) and high purity polysaccharide intercellular adhesin (PIA) isolates were obtained by the protocol of refs 30,43. Briefly, 1L of TSB_G_ media, in 3L Erlenmeyer flasks, was inoculated using 50 mL of overnight culture and incubated for 24–48 h at 60 RPM at 37 °C. Flask adherent biofilm was scraped and the biofilm pellet was collected by centrifugation (4500 *g*, 30 min) and surface attached EPS polymers and PIA was isolated using EDTA as per the protocol of Vuong *et al.*^44^. The extracellular matrix of *S. aureus* biofilms was also purified in this way.

The concentration of glucosaminoglycans in EPS and PIA isolates was measured using the Smith Gilkerson assay^45^. Proteins and nucleic acids in the EPS were quantified using the BCA and PicoGreen assay respectively.

The *in situ* concentrations of PIA, protein, and nucleic acids within *S. epidermidis* biofilms were calculated by measuring the total concentration of EPS polymers per biofilm cell (the latter is quantified as total cell density per culture using a hemocytometer), finding the average extracellular volume available per biofilm cell (from measurements of *in situ* cell number density of Stewart *et al.*^11^) and then taking the ratio the two^30^. The calculated values are averaged over at least 10 different biofilm cultures.

#### Diffusing wave spectroscopy measurements

*S. epidermidis* RP62A biofilms were cultured directly in 1 mm thick rectangular cuvettes by using bacterial colonies to inoculate the TSB_G_ growth media contained in the cuvettes. The colloidal probes were added during inoculation, similar to the work of ref. 15. The cuvettes were then incubated for 24 h at 37 °C at 60 RPM.

#### Quantification of natural biofilm microstructure

Biofilms were grown by passing TSB_G_ through a flow cell with a shear stress of 0.01 Pa, as per ref. 11.

#### Natural biofilms for studying effect of pH on biofilm mechanics

Staphylococcal strains were used to investigate the effect of pH changes on their biofilm mobility and mechanics. These strains were grown in Nunc^TM^ Lab-Tek^TM^ II Chambered Coverglass dishes (Thermo Scientific, USA) for 18 hours at 60 RPM at 37 °C. Each well contained a single bacterial colony and 400 μL TSB_G_ or TSA. After the biofilm containing dishes were removed from the incubator they were stained with LIVE/DEAD (Molecular Probes, Inc., Eugene, OR). Concentrations of 4 μM Syto9 and 25 μM propidium iodide were used. To induce pH changes to the biofilms, the growth media was replaced with 400 μL TSB_G_ with a pH adjusted to 3, 7, or 10. pH was adjusted using 0.3 M acetic acid or 1 M KOH. Media was allowed to incubate with the biofilm at room temperature for 3 hours before imaging occurred.

### Formation of bacterial-chitosan constructs

#### Construct matrix materials: chitosan, bovine serum albumin and DNA

The biofilm matrix was produced in the following way: the PIA was replaced with N-acetylglucosamine glycan chitosan, which differs from PIA in its glycosidic linkages^30^,^46^ and degree of acetylation. Chitosan solution properties such as molar mass and self-associations at acidic pH^30^,^47^ are nearly equivalent to PIA and both complex with proteins and nucleic acids^30^,^48^. The extracellular proteins and DNA were replaced with bovine serum albumin (BSA) and λ-DNA respectively. The chitosan concentration was varied from 0.05 and 0.3 wt. %, representative of the *in situ* PIA concentration in natural biofilms. Because the chitosan dose is prepared at pH = 3.0, this step also introduced pH variation in the artificial biofilms. pH varied in the constructs from 4.3 to 5.7 as chitosan loading was varied from 0.05 to 0.3 wt. % (Supplementary Table S1). pH variations changed the solvent environment of the constructs. BSA and λ-DNA concentrations were determined by the *in situ* stoichiometry of PIA: protein: DNA which is 1:0.5:0.05 (c.f. Supplementary Table S2).

Stock solutions of 1 wt. % chitosan, with manufacturer reported molar mass of 190–300 kDa and a degree of deacetylation of ~75–85% (Sigma Aldrich, St. Louis, MO), were solubilized in 0.3 M Acetic Acid (pH = 3.0). Artificial EPS was prepared in the tryptic soy broth media by mixing together chitosan, BSA (Sigma Aldrich, St. Louis, MO) and λ-DNA (Invitrogen, Eugene, OR) as per refs 30,49 according to their *in situ* stoichiometry.

#### Planktonic bacterial culture for constructs

*S. epidermidis* RP62A was grown in 50 mL TSB_G_ at 200 RPM in an overnight culture in a 250 mL Erlenmeyer flask. 1 mL of the overnight culture was added to 50 mL TSB_G_ and was grown to OD_600_ = 0.5, 1.0, or 1.4. The cellular concentration at each optical density was measured using a Neubauer improved hemocytometer (INCYTO, Korea). Three replicates were performed at each OD and the average and standard error of the mean (SEM) were computed. Bacteria used for formation of constructs were stained with 2.5 μM Syto9 for 30 minutes. Bacterial cellular concentration was varied from 2.4 × 10^8^ to 9.7 × 10^8^ cells/mL (Supplementary Table S3)

#### Artificial *S. epidermidis* biofilms

TSB_G_ was used as the solvent for making the constructs^43^. We varied chitosan concentration between 0.05 and 0.3 wt. %, representative of the *in situ* PIA concentration in natural biofilms. We probed six different chitosan concentrations and three different cellular optical densities. For each *S. epidermidis* cellular OD_600_ value (0.5, 1.0, 1.4), we created bacterial constructs with chitosan concentrations of 0, 0.05, 0.1, 0.2, 0.25, and 0.3 wt. %. Because the chitosan stock solution is prepared at pH = 3.0, this step also introduces pH variation in the artificial biofilms. The pH of the constructs is controlled through this chitosan addition. Chitosan was added to 200 μL *S. epidermidis* cells. Constructs equilibrated for two hours prior to imaging. We decreased the pH of the low-density bacterial constructs (OD_600_ = 1.0, 0.05 wt. % chitosan, pH = 5.3 ± 0.1) to pH = 4.4 by adding 40 μL 0.3 M acetic acid. We increased the pH of the high-density bacterial constructs (OD_600_ = 1.4, 0.3 wt. % chitosan, pH = 4.3 ± 0.02) to 7.3 by adding 18 μL 1 M KOH. We waited 3 hours after the pH was changed before imaging the constructs. The pH range of 4.5 and 6.7 studied here are within the reported pH range of *in situ S. epidermidis* biofilms (4.5–7.5)^32^,^33^,^34^.

### Quantification of *S. epidermidis* RP62A PIA, EPS and biofilm mechanics with particle tracking measurements using diffusing wave spectroscopy (DWS)

The creep compliances of *S. epidermidis* RP62A PIA, EPS and biofilm reported in Fig. 1a were measured using diffusing wave spectroscopy (DWS), a standard technique well suited to rheological characterization in small volumes (~300 μL), as is the case here. DWS was carried out as per ref. 50. Briefly, the probes used were 0.5 μm sulfate latex beads (Invitrogen, Eugene, OR) at concentrations to ensure multiple scattering^50^. The probes were added during inoculation, as in ref. 15. Stability of probes in growth media was confirmed. The mean squared displacement (MSD) of probes in PIA, EPS and biofilm was computed from their normalized intensity autocorrelation function, g_2_(t) = 〈I(0)I(t)〉/〈I(t)〉^2^, where I(t) is the scattering intensity at time t and 〈 〉 is the time average operator, as per ref. 50. The material creep compliance, J(t), was calculated from the MSD following^50^,^51^. We note that, contrary to measurements for the PIA and EPS, DWS measurements of the biofilms exhibited strong probe size dependence. In such cases, DWS measurements measure an apparent rheological property, and a comparison to results from mechanical rheometry benchmark these measurements. In the present case, probes of size 0.5 μm—a dimension comparable to the size of a *S. epidermidis* bacterial cell—yielded a biofilm apparent J(t) that was consistent with that of the mechanical rheometry measurements, whereas measurements at other probe sizes did not (c.f. Supplementary Information). The observed probe size dependence is consist with biofilm structural heterogeneity, and is a further indication of mechanical differences between the cultured biofilms and the PIA and EPS components, which did not show probe size dependent particle dynamics.

### CLSM imaging and analysis of biofilm microstructure and mechanics

The microstructure and mechanical properties of biofilms and bacterial constructs reported in Figs 2, 3, 4 were measured using confocal laser scanning microscopy (CLSM). Bacterial constructs and biofilms were imaged using a Nikon A1Rsi confocal laser scanning microscope with a 100x, 1.45 NA, oil immersion objective lens. Planktonic bacteria used for formation of constructs were stained with 2.5 μM Syto9 for 30 minutes. Natural biofilms were stained with 4 μM Syto9 for 30 minutes. Chitosan was labeled using 10 μg/mL Wheat Germ Agglutinin (WGA), AlexaFluor® 633 (Life Technologies, Grand Island, NY). The excitation wavelength was 488 nm for the Syto9 and 633 nm for the AlexaFluor 633 WGA. Three-dimensional image volumes of size 31 × 31 × 5–23 μm^3^ and time series of 150 two-dimensional images with size 31 × 31 μm^2^ were collected at 15 frames per second.

Local number densities, radial distribution functions (g(r)), and mean squared displacements (MSD) were computed from bacterial centroids identified by previously established image analysis techniques^52^. Bacterial centroids were resolved to within ±35 nm in the object plane and ±45 nm in the axial plane^53^. The lower limit of the instrument sensitivity for the MSD was found to be (Δx^2^(Δt))_min_ = 4.5 × 10^−4^ μm^2^ by tracking fully immobilized bacteria at the coverslip of an arrested sample. There is a small abundance of initially flocculated bacteria in some of the samples (Supplementary Videos 1–5). These flocs are not indicative of the dynamics that determine the construct microrheology and their contribution was subtracted from the measured dynamics (c.f. Supplementary Information). The elasticity (G’) of the constructs was obtained from the MSD by application of 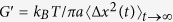, where k_B_ is the Boltzmann constant, T is the temperature, *a* is the bacteria radius and (Δx^2^(Δt)) is the MSD^35^.

### Characterization of polymer phase stability

The phase stability of chitosan and *S. epidermidis* EPS in growth media was studied by measuring absorbance at 600 nm (GENESYS 20, Thermo Scientific, Madison, WI) as a function of pH. The transition from a stable to an unstable phase was denoted as the pH at which a five-fold increase in absorbance was observed.

## Additional Information

**How to cite this article**: Stewart, E. J. *et al.* Artificial biofilms establish the role of matrix interactions in staphylococcal biofilm assembly and disassembly. *Sci. Rep.* **5**, 13081; doi: 10.1038/srep13081 (2015).

## Supplementary Material

Supplementary Video 1

Supplementary Video 2

Supplementary Video 3

Supplementary Video 4

Supplementary Video 5

Supplementary Video 6

Supplementary Video 7

Supplementary Video 8

Supplementary Video 9

Supplementary Information
