# Supplementary material for: Artificial biofilms establish the role of matrix interactions in staphylococcal biofilm assembly and disassembly
Source: Sci Rep. 2015 Aug 14;5:13081. doi: 10.1038/srep13081 (PMC4536489; doi:10.1038/srep13081)
Supplement: Supplementary Information [file srep13081-s10.pdf]

SUPPLEMENTARY INFORMATION

Artificial biofilms establish the role of matrix interactions in staphylococcal biofilm assembly and disassembly

Elizabeth J. Stewart[a,1], Mahesh Ganesan[a,1], John G. Younger[b,2], Michael J. Solomon[a,2]

Author Affiliations:

[a]Department of Chemical Engineering, University of Michigan, 3074 H.H. Dow, 2300 Hayward Street, Ann Arbor, MI 48109

[b]Department of Emergency Medicine, University of Michigan, North Campus Research Complex, 2800 Plymouth Road, Ann Arbor, MI 48109

[1] E.J.S. and M.G. contributed equally to this work.

[2] To whom correspondence and request for materials may be addressed. Email: mjsolo@umich.edu or jyounger@med.umich.edu

SUPPLEMENTARY METHODS

Screening for biofilm-forming clinical isolates of *S. epidermidis.*

To identify biofilm forming clinical isolates, we screened the 54 patient samples of the library of Sharma et al. [1]. For this, we grew all 54 isolates in 10 mL tryptic soy broth with 1 wt. % glucose (TSB_G) at 60 RPM and 37°C overnight in 15 mL culture tubes and visually inspected the walls of the container for biofilm growth. We found 6 potential strains (P2, P6, P12, P18, P37, P47) that were further evaluated for biofilm formation by growing a single colony in 400 μL TSB_G for 18-hours at 60 RPM and 37°C. CLSM was used to inspect for strong biofilm formation, as determined by visual observation of arrested cells within the 18-hour biofilm. We identified *S. epidermidis* P18, P37, and P47 as suitable strains.

Effect of probe size and probe surface chemistry on diffusing wave spectroscopy (DWS) measurements

A probe size and surface chemistry study on PIA and EPS solutions harvested from biofilms indicated no local heterogeneity and no probe-polymer interaction in these solutions. DWS microrheology of the biofilms however exhibited strong probe size dependence. Probes larger than the diameter of *S. epidermidis* cells (> 0.5 μm) exhibited nonergodic dynamics, as characterized by a $g_2(t)$ intercept << 1 [2], because of entrapment within or between biofilm clusters, and were therefore not analyzed further. Probes of size equivalent to that of the bacterial cells – 0.5 μm diameter – exhibited decay in $g_2(t)$ that was consistent with thermally induced random motion. The $g_2(t)$ of probes smaller than the cellular diameter (< 0.5 μm) decayed to a non-zero plateau; this behavior indicates probe localization because of their

entrapment within the biofilm. The biofilm creep compliance, J(t), as a function of probe size is shown in Supplementary Figure 3. We found that the short time response ($t < 10^{-3}$s) was independent of probe size while the long time regime ($t \geq 10^{-3}$ s) was found to be strongly dependent on probe size. Probes of size 0.5 μm – a dimension equivalent to the size of a *S. epidermidis* bacterial cell – resulted in a biofilm J(t) that matched the mechanical rheometry measurements [3].

Microscopic visualization of the probes in the biofilm showed that 0.5 μm carboxylate and amine probes strongly associated with the biofilms (fraction of aggregated or stuck probes > 70%), while 0.5 μm sulfate probes showed much weaker association of < 15%. Using the theory of multiple scattering in binary suspensions [4] and the structure factor formulation for aggregates [5], we found that the impact of this association caused < 1 % change on the mean free path of multiply scattered light and a < 6 % change in the calculated MSD [6].

Accounting for initially flocculated planktonic bacteria in artificial biofilms

We accounted for the presence of flocs in the planktonic cultures used for CLSM microrheology in two ways. First, the 1% of trajectories that were most immobilized at each condition probed were discarded from further analysis. Second, the error in the average $\langle\Delta x^2(\Delta t)\rangle$ due to the flocs within the initial cultures was estimated. To determine this error, we resolved the van Hove self-correlation function of displacement [7] into initially flocculated (slow displacement dynamics) and singlet bacterial (fast displacement dynamics) contributions. We performed this operation for ten different time points of three samples, one from each optical density, as per the methods of Hsiao et al [8]. From the Gaussian distributions of the slow and fast bacterial populations, we estimated $\langle\Delta x^2(\Delta t)\rangle$ for each population. We then compared the slow

and fast bacterial populations to the average $<\Delta x^2(\Delta t)>$ for of the total population. The average $<\Delta x^2(\Delta t)>$ only increased by a factor of four when solely the fast bacteria were accounted for, while the $<\Delta x^2(\Delta t)>$ of the slow bacteria was less than the average $<\Delta x^2(\Delta t)>$ by a factor of 15. Thus, the MSD of the fast (singlet) bacteria is representative of the sample behavior and the results are weakly affected by presence of flocs in the initial bacterial culture. We therefore plot the $<\Delta x^2(\Delta t)>$ of the fast bacterial subpopulation in Figs. 3a, 4c, and 4e.

SUPPLEMENTARY FIGURES

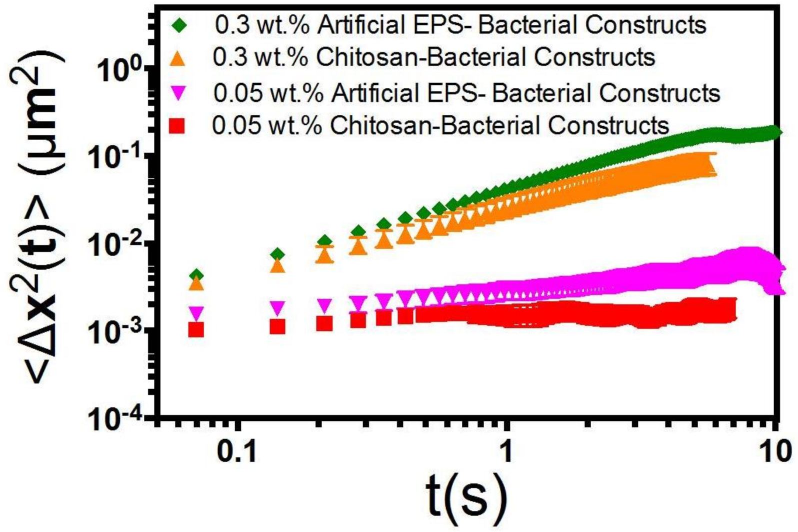

Supplementary Figure S1. MSD of artificial EPS Constructs. The $\langle \Delta x^2(\Delta t) \rangle$ of the constructs with 0.3 wt.% chitosan and the 0.3 wt.% chitosan with λ-DNA and BSA (or artificial EPS) constructs are similar to one another as is the case with the 0.05 wt.% chitosan and 0.05 wt.% artificial EPS. Thus, the effects of the λ-DNA and BSA are small compared to that of the chitosan.

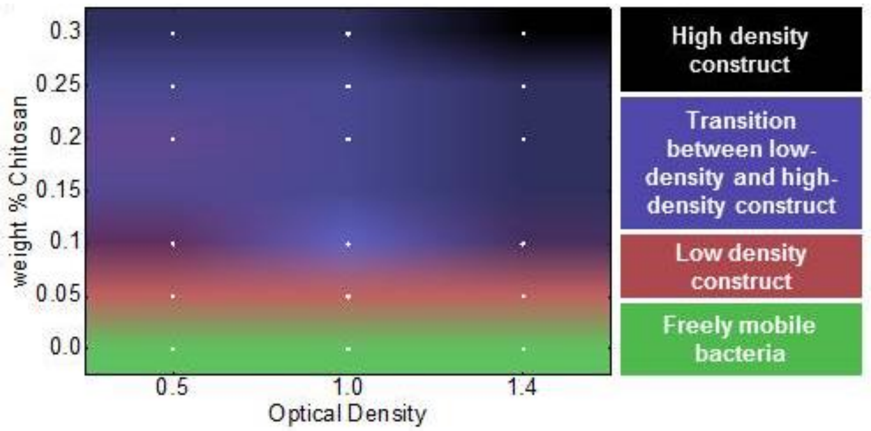

Supplementary Figure S2. Dynamics of constructs at varying cellular and chitosan concentrations. Phase diagram of construct mobility, per visual observation, in which three dynamical regimes exist. The first regime is that of fully mobile planktonic bacteria; the second is the arrested state of the 0.05 wt.% chitosan constructs; the third is a slowed state, present between 0.1 and 0.25 wt.% in which dynamics are intermediate to the planktonic and arrested states.

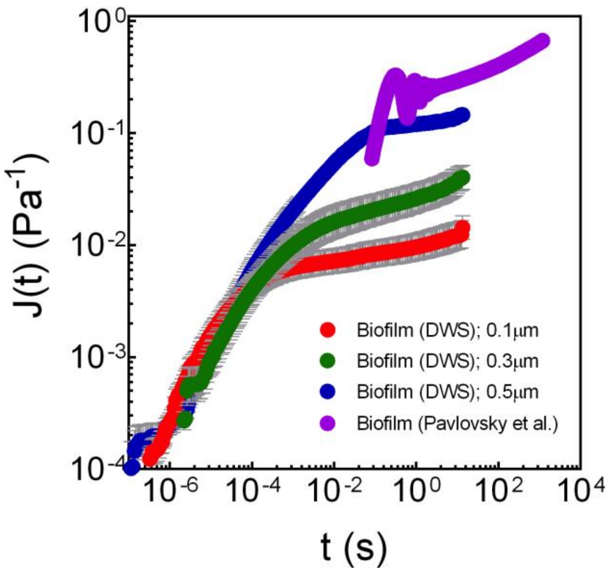

Supplementary Figure S3. Biofilm Creep Compliance. Creep compliance, J(t), of biofilms obtained from DWS microrheology using probes of size 0.1 μm, 0.2 μm and 0.5 μm and from the biofilm mechanical rheometry [3]. The concentration of probes was chosen to ensure multiple scattering following [9].

Supplementary Table S1: pH of bacterial constructs at each OD_{600} and chitosan concentration.

Chitosan (wt.%)	OD_{600} (Cell Concentration- cells/mL)		
	0.5 (2.4E8±0.3E8)	1 (5.1E8±1E8)	1.4 (9.7E8±0.5E8)
0	6.7±0.03	6.3±0.05	5.5±0.2
0.05	5.7±0.03	5.4±0.04	4.9±0.1
0.1	5.1±0.003	5.1±0.2	4.7±0.04
0.2	4.6±0.02	4.6±0.02	4.5±0.03
0.25	4.5±0.01	4.5±0.02	4.4±0.02
0.3	4.4±0.003	4.4±0.01	4.3±0.02

Supplementary Table S2. Average extracellular concentrations, as defined by Ganesan et al.[10] of PIA, protein and DNA within *S. epidermidis* biofilms.

Component	Concentration (g/mL) x 10^2
PIA	2.0 ± 0.5
Total Protein	1.0 ± 0.4
Total DNA	0.1 ± 0.07

Supplementary Table S3. Conversion of optical densities to cell concentrations.

OD_{600}	Cell Concentration (cells/ mL)
0.5	2.4E8 ± 0.3E8
1.0	5.1E8 ± 1E8
1.4	9.7E8 ± 0.5E8

SUPPLEMENTARY VIDEO CAPTIONS

Supplementary Video 1

Planktonic bacterial cells. Freely mobile *S. epidermidis* bacterial cells without chitosan present. Cells are stained with Syto9. Scale bar is 5 µm.

Supplementary Video 2

High cellular density mobile bacterial construct. A high cellular density bacterial-chitosan construct created with cells of OD=1.4 and 0.3 wt. % chitosan at pH = 4.3. The cells are not localized and the chitosan is stable. Scale bar is 5 µm.

Supplementary Video 3

Low cellular density arrested bacterial construct. A low cellular density bacterial-chitosan construct created with cells of OD=1.0 and 0.05 wt. % chitosan at pH = 5.3. The constructs are arrested as a result of chitosan instability. Scale bar is 5 µm.

Supplementary Video 4

High cellular density arrested bacterial construct. A high cellular density bacterial-chitosan construct created with cells of OD=1.4 and 0.3 wt. % chitosan where the pH was increased from 4.3 to 7.3. The chitosan becomes unstable due to the increase in pH, which in turn creates arrested constructs. Scale bar is 5 µm.

Supplementary Video 5

Low cellular density mobile bacterial construct. A low cellular density bacterial-chitosan construct created with cells of OD=1.0 and 0.05 wt. % chitosan where the pH was decreased from 5.3 to 4.4. The chitosan begins to solubilize due to the decrease in pH, resulting in increased mobility of the construct bacteria. Scale bar is 5 µm.

Supplementary Video 6

***S. epidermidis* biofilm control.** *S. epidermidis* RP62A biofilm grown for 18-hours at 60 RPM. The cells within the biofilm are localized and the biofilm has a pH of 5.0. Scale bar is 5 µm.

Supplementary Video 7

***S. epidermidis* biofilm mobilizes after pH increase.** *S. epidermidis* RP62A biofilm where the pH of the media surrounding the biofilm was increased to 7.3 after the biofilm was grown for 18-hours. After four hours, the cells within the biofilm begin to become mobile. Scale bar is 5 µm.

Supplementary Video 8

***S. aureus* biofilm control.** *S. aureus* SH1000 biofilm grown for 18-hours at 60 RPM. The cells within the biofilm are localized and the biofilm has a pH of 4.6. Scale bar is 5 µm.

Supplementary Video 9

***S. aureus* biofilm mobilizes after pH increase.** *S. aureus* SH1000 biofilm where the pH of the media surrounding the biofilm was increased to 6.9 after the biofilm was grown for 18-hours. After four hours, the cells within the biofilm begin to become mobile. Scale bar is 5 μm.

References

1. Sharma, P. et al. Multilocus Sequence Typing for Interpreting Blood Isolates of *Staphylococcus epidermidis*. *Interdiscip Perspect Infect Dis.* **2014,** 787458 (2014).
2. Scheffold, F., Skipetrov, S. E., Romer, S. & Schurtenberger, P. Diffusing-wave spectroscopy of nonergodic media. *Phys Rev E.* **63**, 061404 (2001).
3. Pavlovsky, L., Younger, J. G. & Solomon, M. J. In situ rheology of *Staphylococcus epidermidis* bacterial biofilms. *Soft Matter* **9**, 122-131 (2013).
4. Pine, D. J. et al. in *Scattering and Localization of Classical Waves in Random Media* (ed Sheng, P.) 312-372 (World Scientific, 1990).
5. Sinha, S. K., Freltoft, T. & Kjems, J. in *Kinetics of Aggregation and Gelation* (eds Family, F. & Landau, D. P.) 87-90 (Elsevier, Amsterdam, 1984).
6. Kaplan, P. D., Yodh, A. G. & Pine, D. J. Diffusion and structure in dense binary suspensions. *Phys Rev Lett.* **68**, 393 (1992).
7. Kegel, W. K. & van Blaaderen, A. Direct observation of dynamical heterogeneities in colloidal hard-sphere suspensions. *Science* **287**, 290-293 (2000).
8. Hsiao, L. C., Kang, H., Ahn, K. H. & Solomon, M. J. Role of shear-induced dynamical heterogeneity in the nonlinear rheology of colloidal gels. *Soft Matter* **10**, 9254-9259 (2014).
9. Lu, Q. & Solomon, M. J. Probe size effects on the microrheology of associating polymer solutions. *Phys Rev E.* **66**, 061504 (2002).
10. Ganesan, M. et al. Molar mass, entanglement, and associations of the biofilm polysaccharide of *Staphylococcus epidermidis*. *Biomacromolecules* **14**, 1474-1481 (2013).
